# ABO and Rhesus blood group distribution and frequency among blood donors at Kilimanjaro Christian Medical Center, Moshi, Tanzania

**DOI:** 10.1186/s13104-017-3037-3

**Published:** 2017-12-16

**Authors:** Ola Jahanpour, Jeremia J. Pyuza, Ernest O. Ntiyakunze, Alex Mremi, Elichilia R. Shao

**Affiliations:** 1 School of Public Health, Kilimanjaro Christian Medical University College, P.O. Box, 2240, Moshi, Kilimanjaro Tanzania; 2 Department of Internal Medicine, Kilimanjaro Christian Medical Centre, Longuo B, Sokoine Road, P.O. Box, 3010, Moshi, Kilimanjaro Tanzania; 3 Kilimanjaro Christian Medical University College, P.O.Box 2240, Moshi, Tanzania; 4 Department of Clinical Laboratory, Kilimanjaro Christian Medical Centre, P.O. Box 3010, Moshi, Kilimanjaro Tanzania; 5 Department of Clinical Pathology, Kilimanjaro Christian Medical Centre, P.O. Box 3010, Moshi, Kilimanjaro Tanzania; 6 School of Public Health, Catholic University of Health and Allied Sciences, Bugando Area, P.O. Box 1464, Mwanza, Tanzania; 7 Better Human Health Foundation, P.O. Box 1348, Moshi, Kilimanjaro Tanzania

**Keywords:** ABO, Rhesus factor, Blood donation, Transfusion medicine, Tanzania

## Abstract

**Objective:**

This study aims to determine the distribution of blood groups and the demographic background of blood donors in a referral hospital in Northern Tanzania.

**Results:**

The most common blood group was O (52.3%) and the least common was AB (3.18%). 97.7% of the blood donors were Rh positive and the rest were Rh negative. Most donors were young adults, representing the age group of 19–29. The majority of donors were male (88.1%) and the majority (90.8%) were replacement while the remainder was voluntary donors.

## Introduction

In 1900, Karl Landsteiner of the University of Vienna identified that red blood cells contain antigens on their surfaces, and that blood plasma contains antibodies targeted to particular antigens [1–3]. This discovery is the basis of modern-day blood grouping and transfusion medicine. Demand for whole blood and blood products is high in Tanzania where current supply does not meet the need. Post-partum hemorrhage is a major cause of maternal mortality in Tanzania and lack of adequate blood supply for transfusion is one of the contributing factors [4]. As well, there is a high rate of road traffic injuries, infectious disease such as HIV, gastrointestinal bleeds, and an increasing rate of elective surgeries [4, 5]. Blood banks require timely information concerning the distribution and frequency of blood groups in order to ensure adequate supply of the most medically useful blood types.

Red blood cells antigens, which are the basis of blood grouping, consist of proteins and carbohydrates attached to lipids or proteins. There are more than 100 blood group systems involving over 500 antigens in which ABO is the most studied group in the human population [6]. These antigens have various functions, such as membrane structural integrity and transportation of molecules through membranes. ABO antigens are highly expressed on human tissues and most epithelial and endothelial cells [7, 8]. Antigen expression can influence the development of particular infections as well as certain malignancies. The Multinational Pancreatic Cancer Consortium successfully identified susceptibility loci in the ABO gene for pancreatic cancer pathogenesis [9]. Other studies have showed an association between gastric cancer and blood group A related to a higher susceptibility of Helicobacter pylori infection. Different hypothetical models such as inflammation, immune system surveillance and cell membrane signaling have been developed to explain the mechanism of cancer susceptibility among people with varying blood groups [10–15].

The knowledge of the distribution of Rhesus antigen in a population is critical in managing a transfusion service in areas such as antenatal serology, paternity testing as well as selecting compatible blood and blood products. Even after Karl Landsteiner’s discovery in 1900, transfusion reactions were still prevalent [1]. It was not until 1940 when Landsteiner and Weiner discovered the Rh factor that transfusion medicine involved less risk. Immunogenicity of the Rh factor along with A, B antigens made it mandatory for pre-transfusion testing [1, 16]. Currently there are more than 50 antigens in the Rh blood group system but the principal Rh antigens of medical interest are D, C, E, c and e [16]. A person with Rhesus antigen is referred to as Rhesus positive while individuals lacking the antigen are Rhesus negative. When a Rhesus negative person is exposed to Rhesus positive blood, antibodies will be produced, which cause potentially fatal hemolytic reactions. There is a lack of published data in Tanzania describing the distribution of ABO and Rh among blood donors, and this information is scarce in Africa as a whole [17, 18]. This study aims to determine the distribution of blood groups and the demographic background of blood donors in a referral hospital in Northern Tanzania.

## Main text

### Methods

A cross-sectional study was conducted at KCMC Clinical Laboratory in the blood transfusion unit over a period of 6 months from October 2014 to March 2015. Samples were analyzed from both voluntary and replacement donors at KCMC Clinical Laboratory, Moshi, Tanzania collected from October 2013 to September 2014. Voluntary blood donation involves a donor giving blood, plasma or cellular components of his or her own free will while replacement or family donors are those who give blood when required by a family or community member [19].

The inclusion criteria for this study were donors between the ages of 18–70, with a personal weight above 50 kg, and who met the hemoglobin cut off criteria. All donors were required to have a haemoglobin level of at least 12.0 g/dL for females and 13.0 g/dL for males as per WHO standards. A total of 1845 participants out of 2200 total donors met inclusion criteria. 1815 participants (98.4%) had the complete information required for analysis in this study.

During blood donation approximately 4 mL of blood from each donor was collected in EDTA tubes for analysis. ABO and Rh status were analyzed by tube method using commercially prepared anti-A, anti-B, anti-AB and anti-D antisera blood types. To do so correctly, we followed the specific procedures outlined in the manufacturer’s manual. Prepared 5% suspensions of red blood cells in normal saline were used. Four different tubes labelled with donor unit numbers were added with one drop of antisera A, B, AB and D. To every tube with specific antisera one drop of 5% cell suspension was added and each sample was macroscopically observed for agglutination.

Descriptive statistics was used whereby data were summarized using frequency and percentages. Fisher’s Exact test was used to test association between exposures and outcome of interest. A *P value* of less than 0.05 (2-tails) was considered as statistically significant.

### Interpretation of results

Positive: Agglutination indicates positive reactions to respective group or Rhesus factor.

Negative: No Agglutination indicates negative reactions to respective group or Rhesus factor (Table 3).

### Results

A total of 1845 participants met inclusion criteria. Out of the total participants, there was complete information for 1815 (98.4%) blood donors. As shown in Table 1, there were more male participants (88%, n = 1597) as compared with female participants (12%, n = 218). The age distribution of the participants was 43% (n = 773), 29% (n = 530), and 28% (n = 508) for the age groups of 18–29, 30–39 and 40–65 respectively.

**Table 1:** Gender and age distribution of the participants (N = 1815)

Characteristic	Frequency (n)	Percentage (%)
Sex		
Female	218	12
Male	1597	88
Age (years)		
18–29	773	43
30–39	530	29
40–65	508	28

The most common blood type among the participants was blood group O (52%, n = 949), followed by blood group A (26%, n = 465), blood group B (19%, n = 342) and blood group AB (3%, n = 59%). As shown in Table 2, 98% (n = 1773) of participants were Rhesus positive while 2% (n = 42) were Rh negative.

**Table 2:** Blood type and Rh antigens of the participants (N = 1815)

	Frequency n (%)
Blood type	
A	465 (26)
B	342 (19)
O	949 (52)
AB	59 (3)
Rh antigens	
Rh negative	42 (2)
Rh positive	1773 (98)

In both sexes, blood group O was the commonest being 52% (n = 830) among male and 55% (n = 119) among female participants. The least common blood group was blood group AB representing 3% of male participants and 1.8% of female participants. There was no difference between the blood type and the sex (χ^2^ = 3.7021, *P value* 0.895). Except for blood group AB, all of the other blood groups had Rh-negative antigens in the donated blood. The prevalence of Rh-negative antigens were 3% (n = 13), 2% (n = 8) and 2% (n = 21) for blood group A, B and O respectively. There were no differences in RH between different blood groups (χ^2^ = 1.923, *P value* 0.712) (Table 3).

**Table 3:** Distribution of Rh antigens per ABO blood group among participants (N = 1815)

	Rh −ve n (%)	Rh +ve n (%)
A	13 (3)	452 (97)
B	8 (2)	334 (98)
O	21 (2)	928 (98)
AB	–	59 (100)

ABO and Rhesus blood groups distribution among the first time blood donors at KCMC referral and teaching hospital in Northern Tanzania

### Discussion

This study determined the distribution of ABO and Rh antigens among blood donors in Northeastern Tanzania, where there is a lack of data on this subject [17, 18]. The current study showed that the majority of donors were male, which is consistent with other studies in Africa and in most regions globally [17–19, 20, 22, 23]. One contributing factor might be that women do not meet donation cut-off values for hemoglobin given normal menses, menorrhagia, prenatal iron deficiency anemia and postnatal blood loss. From a cultural perspective, in various African countries it may be more likely for males to donate blood given long-standing beliefs that women are not as physically strong as men [22, 23]. In Western regions such as Europe, women were found to have higher rates of adverse reactions, primarily vasovagal events, and were also not as likely to meet hemoglobin cut off requirements for donation [23].

In studies spanning diverse cultural and geographic groups, the most common age range for blood donation is 18–44 years [24]. Our study results are consistent with this global trend. The improved interest and ability among younger adults to donate may be related to awareness, better physical health, and greater mobility. Older individuals may suffer from medical conditions such as ischemic heart disease, diabetes mellitus, malignancy and hypertension hence negatively impacting their ability to be well enough to donate blood. There is a need to better understand the potential of adolescents to contribute [25], however, which was not addressed in this study.

The majority of donors in our study were replacement (90%) while the minorities were voluntary (10%). This is consistent with other studies and global trends [19, 21, 26]. The donation of blood by voluntary non-remunerated blood donors is critical for the safety and sustainability of national blood supplies. National blood donation systems in which replacement donors dominate are typically unable to meet clinical demands for blood while paid family members contributing often poses serious threats to the health and safety of the recipients and the donors. WHO recommendations are therefore to create health systems based 100% on voluntary donation [19].

The predominant blood group in our study was type O and the least common was AB. This is consistent with studies in Nigeria, which also showed the predominant group to be O and the least common to be AB [27]. In Kenya, Uganda, Mauritania and Ethiopia similar studies also showed the predominant blood group to be O and the least prevalent to be AB [27–30]. These trends, in keeping with other studies, may suggest that blood group AB is the least dominant while O is the most dominant overall across the continent.

However, there is regional variability; some studies show that in Western and Central Africa, the most predominant group was B while in Eastern and Southern countries, blood group O dominated [31–33]. In Pakistan, one study showed that blood group B is the most dominant while in Nepal it was blood group B. In Britain the most predominant blood group is O and the least is AB [31]. These regional differences may be explained by genetic mapping and the varying origins of diverse ethnic groups [31].

Determination of Rh status is crucial in clinical contexts in order to ensure patient safety. Rh factor is of interest because of its marked immunogenicity. In the case of the D antigen, patients who do not produce the D antigen will produce anti-D if they encounter the D antigen on transfused red blood cells. This process may result in a hemolytic transfusion reaction or, in the case of newborn red blood cells, hemolytic disease of the newborn [31]. For this reason, it is important to determine the Rh status in clinical settings and for research purposes.

Individuals who are Rhesus negative in our study were only 2.3% in contrast to other studies, which showed a range between 5 and 17%. Our study showed a slightly lower prevalence of Rh positive blood donors in comparison to other studies in the African continent as well as in comparison with global trends [34–36]. Given the number of participants in this study, however, this cannot be said to be statistically significant. Globally we share the same blood group types however clearly there are some geographic, regional, and ethnic differences. Ensuring adequate Rh positive blood supply is important in the context of patient safety. As well, the growing literature investigating the association of blood groups with the pathogenesis of cancer requires locally specific information on Rh distribution among other factors [37].

### Conclusion

Up-to-date knowledge of the distribution of blood types in a local setting is critical to the functioning of any national health service. To date, there has been a lack of data on this important topic in Tanzania. Our study provides detailed information concerning the blood type and demographic information of blood donors in the Northeastern region of Tanzania. Similar studies are needed across the country and further research and mobilization are required to meet WHO recommendations on voluntary blood donation.

## Limitations

This study was conducted in the Northeastern region of Tanzania; the results should not be generalized to Tanzania as a whole.

## Abbreviations

Rh: Rhesus
RTI: road traffic injuries
KCMC: Kilimanjaro Christian Medical center
WHO: World Health Organization
